# Health-related quality of life of X-linked hypophosphatemia in Spain

**DOI:** 10.1186/s13023-022-02452-0

**Published:** 2022-07-29

**Authors:** M. I. Luis Yanes, M. Diaz-Curiel, P. Peris, C. Vicente, S. Marin, M. Ramon-Krauel, J. Hernandez, J. J. Broseta, L. Espinosa, S. Mendizabal, L. Perez-Sukia, V. Martínez, C. Palazón, J. A. Piñero, M. A. Calleja, J. Espin, R. Arborio-Pinel, G. Ariceta

**Affiliations:** 1 Hospital Nuestra Señora de Candelaria, Santa Cruz de Tenerife, Spain; 2 Fundación Jiménez Díaz, Madrid, Spain; 3 Hospital Clínic, Barcelona, Spain; 4 Hospital Virgen de Arrixaca, Murcia, Spain; 5 Hospital Sant Joan de Déu, Esplugues de Llobregat (Barcelona), Spain; 6 Hospital La Fe, Valencia, Spain; 7 Hospital La Paz, Madrid, Spain; 8 Hospital Virgen de la Macarena, Seville, Spain; 9 Escuela Andaluza de Salud Pública, Granada, Spain; 10 Hospital Vall d’Hebron, Barcelona, Spain

**Keywords:** Burden of disease, X-linked hypophosphatemia, Health-related quality of life, EQ-5D

## Abstract

**Background:**

Health-related quality of life (HRQoL) of patients with X-linked hypophosphatemia (XLH) is lower than that of both the general population and the patients with other chronic diseases, mainly due to diagnostic delay, treatment difficulties, poor psychosocial support, and problems with social integration. Early diagnosis and optimal treatment are paramount to control the disease in patients with XLH, avoid complications, and maintain or improve their HRQoL. We, therefore, analyzed the HRQoL of pediatric and adult patients with XLH treated with conventional therapy in Spain.

**Results:**

We used several versions of the EuroQol-5 dimensions (EQ-5D) instrument according to the age of patients with XLH. Then we compared the HRQoL of patients to that of the general Spanish population. Children with XLH (n = 21) had moderate problems in walking about (61.9%), washing or dressing themselves (9.52%), and performing their usual activities (33.33%). They also felt moderate pain or discomfort (61.9%) and were moderately anxious or depressed (23.81%). Adults with XLH (n = 29) had lower HRQoL, with problems in walking (93%, with 3.45% unable to walk independently), some level of pain (86%, with 3.45% experiencing extreme pain), problems with their usual activities (80%) and self-care (> 50%), and reported symptoms of anxiety and/or depression (65%). There were important differences with the general Spanish population.

**Conclusions:**

XLH impacts negatively on physical functioning and HRQoL of patients. In Spanish patients with XLH, the HRQoL was reduced despite conventional treatment, clearly indicating the need to improve the therapeutic approach to this disorder.

**Supplementary Information:**

The online version contains supplementary material available at 10.1186/s13023-022-02452-0.

## Plain English summary

X-linked hypophosphatemia (XLH) is a severe inherited disease. It is caused by loss of phosphorus by kidneys. As a result, blood level of phosphorus is low, affectingX-linked hypophosphatemia (XLH) is a severe inherited disease. It is caused by loss of phosphorus by kidneys. As a result, blood level of phosphorus is low, affecting bones and muscles. Patients can have growth retardation, short stature, rickets, limb deformities, pain and other health problems despite traditional treatment. Consequently, their quality of life can be very bad. However, a recently available new treatment (burosumab) can improve this quality of life. We studied the quality of life of children and adults with XLH treated with traditional treatment in Spain. Results showed that children had moderate problems, but adults reported moderate-to-severe problems in walking and performing their usual activities and self-care. Pain and anxiety and/or depression were very frequent. There were important differences with the general Spanish population. Moreover, we also found that XLH is associated to high healthcare cost and even higher socioeconomic cost. Our results highlight the need of improving the treatment of XLH.bones and muscles. Patients can have growth retardation, short stature, rickets, limb deformities, pain and other health problems despite traditional treatment. Consequently, their quality of life can be very bad. However, a recently available new treatment (burosumab) can improve this quality of life. We studied the quality of life of children and adults with XLH treated with traditional treatment in Spain. Results showed that children had moderate problems, but adults reported moderate-to-severe problems in walking and performing their usual activities and self-care. Pain and anxiety and/or depression were very frequent. There were important differences with the general Spanish population. Moreover, we also found that XLH is associated to high healthcare cost and even higher socioeconomic cost. Our results highlight the need of improving the treatment of XLH.

**Supplementary Information:**

The online version contains supplementary material available at 10.1186/s13023-022-02452-0.

## Background

Health-related quality of life (HRQoL) in patients with rare diseases and their caregivers has not been widely studied [1]. However, in a study of 1218 adults with different rare diseases, HRQoL was found to be lower than that of both the general US population, and patients with common chronic diseases [2]. Low HRQoL in rare diseases is caused by diagnostic delay, treatment difficulties, poor psychosocial support, and problems with social integration [2]. In a survey in Spain of 1576 patients with rare diseases, 44.64% of patients reported unsatisfactory HRQoL and 7.64% considered that their HRQoL was very bad [3].

X-linked hypophosphatemia (XLH) is a severe, debilitating and deforming condition. It is a rare disease, with a prevalence of 1:20,000 [4]. XLH is the most common form of hereditary hypophosphatemia and is caused by inactivating mutations in the Phosphate Regulating Endopeptidase Homolog X-Linked (*PHEX*) gene [5]. These mutations produce increased levels of fibroblast growth factor 23 (FGF23), a phosphate-regulating hormone that seems to mediate most manifestations of the disease [4]. Elevated levels of FGF23 decrease renal reabsorption of phosphate by inhibition of sodium-phosphate transporters in kidneys, thus increasing urinary phosphate excretion, and limit intestinal phosphate absorption by reducing calcitriol (1,25(OH)_2_D) synthesis, whose levels are low or even normal despite the hypophosphatemia [4, 6]. Low levels of phosphate and calcitriol are related to many pathologic changes of XLH [4]. Symptoms are numerous and variable, with different levels of severity [7], and the onset is usually during the first or second year of life [8, 9]. Disease manifestations in children include impaired growth [10] with short stature [7] and bone deformities [8, 9], especially in lower limbs [7], and radiological signs of active rickets [8, 9]. Moreover, pain [11], poor mineralization of teeth [11] and craniosynostosis [12] have also been described. Adult patients with XLH also have skeletal pain and significant associated morbidity due to long-term complications such as osteoarthritis, osteomalacia, enthesopathies, spinous ligament calcification or spontaneous insufficiency fractures [7], among others. These complications clearly worsen the HRQoL of patients [7].

Burden of XLH is substantial, both in terms of impairment of HRQoL and of direct and indirect healthcare costs. Taking into account the aforementioned, early diagnosis and optimal treatment are paramount to control the disease in patients with XLH, avoid complications, and maintain or improve their HRQoL, and therefore to decrease the associated economic burden of the disease [13]. There are few studies related to HRQoL in patients with XLH, either children [14, 15] or adults [16, 17]. We therefore analyzed the HRQoL of patients with XLH in Spain in order to improve the care of patients with this rare disease.

## Results

The study was conducted from September 2017 to May 2018, with patient inclusion carried out from December 2017 to April 2018. Fifty patients participated in the study: 21 children (< 18 years) accompanied by their respective parents or caregivers, and 29 adults. These latter were 21 women and 8 men, with a mean age of 42.21 ± 16.18 years, and with 5 patients (all women) aged 60 years or older.

### Quality of life in pediatric patients with XLH

The results of EuroQol-5 dimensions three-level version (EQ-5D-3L) and EQ-5D-3L proxy questionnaires (Table 1) showed that more than 60% of children had mobility difficulties. Although less than 10% of children had some difficulties with self-care, almost 34% had difficulties performing their usual activities, more than 60% reported pain or discomfort, and nearly 24% felt anxious or depressed.

**Table 1:** Quality of life in children with X-linked hypophosphatemia (n = 21). Response percentages in the different dimensions of the EQ-5D-3L and EQ-5D-3L proxy questionnaires

Dimension	Children (n = 21) n (%)
**Mobility**	
No problems	8 (38.10%)
Moderate problems	13 (61.90%)
Unable to walk about	0 (0.00%)
**Self-care**	
No problems	19 (90.48%)
Moderate problems	2 (9.52%)
Unable to wash or dress himself/herself	0 (0.00%)
**Usual activities**	
No problems	14 (66.67%)
Moderate problems	7 (33.33%)
Unable to do usual activities	0 (0.00%)
**Pain/discomfort**	
No pain or discomfort	8 (38.10%)
Moderate pain or discomfort	13 (61.90%)
Extreme pain or discomfort	0 (0.00%)
**Anxiety/depression**	
Not anxious or depressed	16 (76.19%)
Moderately anxious or depressed	5 (23.81%)
Extremely anxious or depressed	0 (0.00%)

The mean EQ-5D-3L (and EQ-5D-3L proxy) index was 0.788 ± 0.153 and the mean visual analog scale (VAS) score was 68.33 ± 16.06.

### Quality of life in adult patients with XLH

Mobility was the most affected dimension in adults with XLH, with 93% of patients reporting walking difficulties. More than 50% of patients had difficulties in taking care of themselves, and 80% of patients reported difficulties doing their usual activities. Pain/discomfort was reported by 86% of patients, with severe or extreme pain in more than 40%. In addition, 65% of patients felt some level of anxiety/depression (Table 2). The mean EuroQol-5 dimensions five-level version (EQ-5D-5L) index was 0.562 ± 0.28 and the mean VAS score was 55.96 ± 20.86.

**Table 2:** Quality of life in adults with X-linked hypophosphatemia (n = 29)

Dimension	Adults (n = 29) n (%)
**Mobility**	
No problems	2 (6.90%)
Slight problems	11 (37.93%)
Moderate problems	9 (31.03%)
Severe problems	6 (20.69%)
Unable to walk about	1 (3.45%)
**Self-care**	
No problems	14 (48.28%)
Slight problems	9 (31.03%)
Moderate problems	5 (17.24%)
Severe problems	1 (3.45%)
Unable to wash or dress	0 (0%)
**Usual activities**	
No problems	6 (20.69%)
Slight problems	10 (34.48%)
Moderate problems	9 (31.03%)
Severe problems	4 (13.79%)
Unable to do usual activities	0 (0%)
**Pain/discomfort**	
No pain or discomfort	4 (13.79%)
Slight pain or discomfort	3 (10.34%)
Moderate pain or discomfort	10 (34.48%)
Severe pain or discomfort	11 (37.93%)
Extreme pain or discomfort	1 (3.45%)
**Anxiety/depression**	
Not anxious or depressed	10 (34.48%)
Slightly anxious or depressed	9 (31.03%)
Moderately anxious or depressed	7 (24.14%)
Severely anxious or depressed	3 (10.34%)
Extremely anxious or depressed	0 (0%)

Response percentages in the different dimensions of the EQ-5D-5L questionnaire

On comparing our results with those of the 2011 National Health Survey of Spain (ENSE) [18], adult patients with XLH had poorer HRQoL than the general Spanish population (Additional file 1: Table S1), with differences in all dimensions. In order of difficulty/severity, these differences were mobility, pain/discomfort, usual activities, self-care and anxiety/depression. Mobility difficulties were reported by 93.10% of patients with XLH compared with 14.28% of the general population, including 3.45% and 0.82%, respectively, who were unable to walk. Pain/discomfort was described by nearly 87% of patients compared with 25% of the general population. Differences between patients and the general population were especially marked for moderate and severe pain. Nearly 80% of patients with XLH presented difficulties in performing their usual activities while only 11% of the general population reported difficulties. More than 50% of patients with XLH reported some hardship with self-care compared with only 6% of the general population. Finally, more than 65% of patients with XLH but only 15% of the general population had some level of depression/anxiety. The worse HRQoL in adult patients with XLH was also expressed by the very different mean EQ-5D-5L index: 0.562 ± 0.28 in patients and 0.914 ± 0.15 in the general population [18]. Similarly, the mean VAS score in adult patients with XLH (55.96 ± 20.86) was much lower than that of the general population (77.53 ± 18.60) [18].

Adult patients with XLH had a lower HRQoL than caregivers and parents, as shown in all dimensions of the EQ-5D-5L questionnaire (Additional file 1: Table S2). For caregivers and parents, the EQ-5D-5L index was 0.821 ± 0.157 and the VAS score was 75.47 ± 17.24. These values were slightly lower than those found in the general Spanish population: 0.914 ± 0.15 and 77.53 ± 18.60, respectively [15].

## Discussion

This study showed that burden of XLH in Spain is substantial, as XLH greatly impairs the HRQoL of pediatric and adult patients.

Pediatric patients had difficulties in the five dimensions of the EQ-5D-3L and EQ-5D-3L proxy. More than 60% of pediatric patients had moderate walking difficulties and the same percentage reported moderate pain/discomfort. In an international study, the HRQoL of 90 children with XLH was assessed with the 10-Item Short Form Health Survey (SF-10), that was fulfilled by caregivers or parents. The Physical Health Summary score of the SF-10 showed an impairment of HRQoL with almost one and a half standard deviations below the US general population norm [19]. Although we could not directly compare HRQoL results in pediatric patients with XLH with those of ENSE 2011, we can make an approximation with the results in children from 6 to 17 years old from the general Spanish population that participated in a study of validation of the EQ-5D-Y (child-friendly EQ-5D) instrument [20]. This questionnaire uses a simpler language than the EQ-5D-3L instrument, but questions and answers are basically the same. Impairment of HRQoL in pediatric patients is apparent compared with the general Spanish population, especially in the mobility, usual activities and pain/discomfort domains. For the self-care and mood domains, differences still exist but are not so large (Table 3).

**Table 3:** Differences in pediatric patients with XLH (n = 21) and children and adolescents from general Spanish population (n = 620)

Domain*	Problems	Patients with XLH, %	General Spanish population, %
Mobility	No	38.1	96.8
	Moderate	61.9	2.6
Self-care	No	90.5	96.6
	Moderate	9.5	3.1
Usual activities	No	66.7	92.7
	Moderate	33.3	6.1
Pain/discomfort	No	38.1	84.2
	Moderate	62	15
Mood	No	76.2	79.8
	Moderate	23.8	18.9

*EQ-5D-3L or EQ-5D-Y

Reduction of HRQoL was even more important in adult patients, with bigger impairment in all EQ-5D-5L dimensions. Impairment was especially marked in the mobility dimension, with nearly 25% of patients having severe or extreme problems, and in the pain/discomfort dimension, with 40% of patients having severe or extreme pain. There were also evident difficulties in performing usual activities and self-care, as well as symptoms of anxiety and/or depression. Compared with the Spanish general population [18], adult patients with XLH reported worse results in all EQ-5D-5L dimensions. This questionnaire was also used in a UK study in 24 adult patients with XLH. The scores were higher than those obtained by patients in our study: mean EQ-5D index (using the England value set) 0.648 ± 0.29 and mean VAS score 60.8 ± 26.9 [17]. Reduced HRQoL in adult patients with XLH has been shown in other studies, although using different assessment instruments. In 52 patients, the Health Assessment Questionnaire (HAS), the Routine Assessment of Patient Index 3 (RAPID3) and the 36-item Short Form Health Survey (SF-36) showed an impairment in HRQoL, especially in older patients and those with structural lesions [16]. Moreover, HRQoL of patients with XLH was lower than that of patients with axial spondyloarthritis [16] and similar to that of patients with osteogenesis imperfecta or with fibrous dysplasia [17]. Additionally, it has been confirmed the negative impact of bone and joint pain and functional limitations on wellbeing and quality of life of patients with XLH [21]. Thus, in a recent international study in 232 patients with XLH, pain and functional limitations were the main causes of a HRQoL lower than that of general population [19]. As for caregivers and parents, the mean EQ-5D-5L index and the mean VAS score were slightly lower than that in general Spanish population. In the field of rare diseases, the quality of life of caregivers is still not well investigated.

Differences between HRQoL impairment between pediatric and adult patients might be lower than apparently were. Results of HRQoL instruments in children may not reflect actual quality of life. Concepts of health and disease can be different for children according to their age and cognitive development. Likewise, the effects of the disease and its therapies can be different for children. Therefore, children may not have the same perception of the disease consequences and impact. In addition, EQ-5D questionnaires do not assess factors that can modify the quality of life in children, such as familial relationships or cognitive skills. Thus, whenever possible, it is recommended to use children-specific instruments, as well as disease-specific questionnaires or scales [22]. However, we did not use a children-specific instrument but the EQ-5D-3L or the EQ-5D-3L proxy. We chose the EQ-5D instrument because it has been used in other studies of HRQoL in patients with XLH [17] as well as in patients (mainly children) with other rare diseases [23]. Moreover, the EQ-5D questionnaires are accepted for assessing the HRQoL in children, and the EQ-5D-3L proxy has been considered as a valid instrument [24, 25]. Another, and relevant, reason to choose the EQ-5D instruments was that they are recommended for studies on the use of resources with adults and children for the effects of comparability of the results [22]. Likewise, this type of instruments to quantify the quality of life of patients with XLH are gaining more and more importance in recent years, so they should be valued accordingly [26] for this type of research. Moreover, in a recent meta-analysis of the health impact of rare diseases, the lowest quality of life across EQ-5D scores, VAS scores and Barthel index corresponded to patients with musculoskeletal diseases [27]. Nonetheless, it should be noted that EQ-5D has also been recognized as being inaccurate for measuring quality of life and in particular for rare diseases [28–30].

HRQoL impairment suggests that conventional treatment with oral phosphorus and calcitriol does not prevent the complications of XLH that lead to HRQoL impairment, which is especially relevant in adult patients. The treatment of XLH should act on the disease mechanisms and should be able to modify the natural history of the disease [31, 32]. In this sense, burosumab is an anti-FGF23 fully human monoclonal antibody that has shown to improve many of the XLH complications in children [14, 15, 33, 34] and adults [35–38] increasing their quality of life. Currently, there are no specific patient-reported outcomes (PROs) to measure the improvements in quality of life in patients with XLH. This is in part due to the low prevalence of the disease. However, some efforts have been started in this direction, yet in need of validation within larger populations [39–41]. The phase 3 clinical trial of burosumab in adults with XLH took into consideration the XLH-specific meaningful changes on PROs to evaluate the effect of burosumab on physical function, stiffness, pain, and fatigue [35]. Nevertheless, as indicated above, the validation of these tools in patients with XLH to monitor the quality of life and the related thresholds to categorize changes as clinically meaningful needs to be completed.

The study has some limitations. One of them was the limited sample size due to XLH is a very rare disease. This inconvenience did not allow to analyze potential differences in HRQoL by sex or age groups. However, although the number of patients studied was small, we consider that sample size was sufficiently representative for our study. Another limitation was that there is no specific instrument to assess HRQoL in patients with XLH and we used the generic EQ-5D-5L and EQ-5D-3L instruments. Furthermore, XLH is a variable and heterogeneous disease, and hence statistical analysis could be more inaccurate than in other diseases.

Further investigations included in our research agenda will be performed to evaluate the direct and indirect costs derived from this disease.

## Conclusions

According to our results, we can conclude that XLH impacts negatively on physical functioning and HRQoL of Spanish patients treated with conventional therapy, clearly indicating the need to improve the therapeutic approach of this disorder.

## Methods

The project was the result of the collaboration of a committee of experts on XLH. The main objective of the study was to assess the HRQoL of patients with XLH and their caregivers in Spain. Thus, we carried out a multicenter, cross-sectional observational study of pediatric (< 18 years) and adult (≥ 18 years) patients diagnosed with XLH, managed in specialized clinics of tertiary hospitals across Spain. Parents or caregivers accompanied all pediatric patients.

### HRQoL assessment

HRQoL was assessed with the paper-based Spanish version of the EQ-5D instrument [42], which has been validated in the Spanish population [43]. We used the EQ-5D-3L for patients aged between 12 and 17 years; the EQ-5D-3L proxy, which was completed by parents or caregivers of patients under 12 years; and the EQ-5D-5L, which was given to adults and caregivers. All questionnaires were anonymous, and no personal data was collected. The database was compiled in accordance with the Spanish law on personal data protection (Organic Act 15/1999, of December 13) and the prevailing regulation (Royal Decree 1720/2007, of December 21).

The EQ-5D consists of five questions corresponding to five dimensions (mobility, self-care, usual activities, pain/discomfort, and anxiety/depression) and a VAS [44]. The EQ-5D-3L and EQ-5D-3L proxy questionnaires have three possible answers for each dimension, scored from 1 to 3. Similarly, the EQ-5D-5L questionnaire has five possible answers for each dimension, scored from 1 to 5. This is a descriptive system from which a five-digit health state profile is obtained; the profile expresses the level of difficulty experienced in each of the five health dimensions [44]. A weighted overall score (utility index or EQ-5D index) from 0 (death) to 1 (perfect health) can also be obtained, although negative values are allowed for “worse than death” states. The second part of the EQ-5D instrument is a VAS of health status. The scale scores from 0 to 100, where 0 is the worst possible state and 100 the best possible state [44]. We recorded results of the EQ-5D questionnaires in Excel spreadsheets and then we estimated the response percentages for each question as well as the mean and ± SD for the VAS.

The results were not presented as health profiles but rather as percentages of responses to each option in the five dimensions for pediatric and adult patients. The data collected from the questionnaires was aggregated to estimate the mean ± SD for the EQ-5D index and VAS for pediatric and adult patients as well as for caregivers.

We compared HRQoL results in adult XLH patients with those of the general Spanish population according to the ENSE 2011 [18], which utilized the EQ-5D-5L questionnaire to assess HRQoL in adults. However, quality of life in children and adolescents was not evaluated in this survey.

### Statistical analysis

All data were managed with Microsoft® Excel® Spreadsheet Software version 14.0 (Microsoft, Seattle, WA, USA). A descriptive analysis of the frequency distribution was made for qualitative variables, and an estimation of mean, standard deviation, maximum, minimum and 95% confidence interval was carried out for quantitative variables.

## Supplementary Information

**Additional file 1.** Additional Tables.

## Data Availability

All the data generated or analyzed during this study are included in this article and its Additional file 1.

## Abbreviations

1,25(OH)_2_D: Calcitriol
BPI: Brief pain inventory
ENSE: National Health Survey of Spain
EQ-5D: EuroQol-5 dimensions
EQ-5D-3L: EuroQol-5 dimensions three-level version
EQ-5D-5L: EuroQol-5 dimensions five-level version
FGF23: Fibroblast growth factor 23
HAS: Health Assessment Questionnaire
HRQoL: Health-related quality of life
PRO: Patient-reported outcome
RAPID3: Routine Assessment of Patient Index 3
SF-10: 10-Item Short Form Health Survey
SF-36: 36-Item Short Form Health Survey
VAS: Visual Analog Scale
WOMAC: Western Ontario and McMaster Universities Osteoarthritis Index
XLH: X-linked hypophosphatemia

## References

[1] López-Bastida J, Oliva-Moreno J, Linertova R, Serrano-Aguilar P (2016). Social/economic costs and health-related quality of life in patients with rare diseases in Europe. Eur J Health Econ.

[2] Bogart KR, Irvin VL (2017). Health-related quality of life among adults with diverse rare disorders. Orphanet J Rare Dis.

[3] Federación Española de Enfermedades Raras (FEDER) and the Creer center. [Survey on socio-sanitary needs of people with rare diseases in Spain. ENSERio study. 2016–2017 data]. Madrid [cited 2022 June 1st]. Available from: https://obser.enfermedades-raras.org/wp-content/uploads/2018/12/FINAL-ENSERio_Estudio-sobre-situacion%20de-Necesidades-Sociosanitarias-Personas-con-Enfermedades-Raras-en-Espana.pdf.

[4] Beck-Nielsen SS, Mughal Z, Haffner D, Nilsson O, Levtchenko E, Ariceta G (2019). FGF23 and its role in X-linked hypophosphatemia-related morbidity. Orphanet J Rare Dis.

[5] Francis F, Hennig S, Korn B, Reinhardt R, de Jong P, Poustka A (1995). A gene (*PEX*) with homologies to endopeptidases is mutated in patients with X-linked hypophosphatemic rickets. The HYP Consortium. Nat Genet.

[6] Imel EA, DiMeglio LA, Hui SL, Carpenter TO, Econs MJ (2010). Treatment of X-linked hypophosphatemia with calcitriol and phosphate increases circulating fibroblast growth factor 23 concentrations. J Clin Endocrinol Metab.

[7] Carpenter TO, Imel EA, Holm IA, de Beur SMJ, Insogna KL (2011). A clinicians guide to X-linked hypophosphatemia. J Bone Miner Res.

[8] Rodríguez-Rubio E, Gil-Peña H, Chocron S, Madariaga L, de la Cerda-Ojeda F, Fernández-Fernández M (2021). Phenotypic characterization of X-linked hypophosphatemia in pediatric Spanish population. Orphanet J Rare Dis.

[9] Rodríguez-Rubio E, Gil-Peña H, Chocron S, Madariaga L, de la Cerda-Ojeda F, Fernández-Fernández M (2021). Correction to: Phenotypic characterization of X-linked hypophosphatemia in pediatric Spanish population. Orphanet J Rare Dis.

[10] Fuente R, Gil-Peña H, Claramunt-Taberner D, Hernández O, Fernández-Iglesias A, Alonso-Durán L (2017). X-linked hypophosphatemia and growth. Rev Endocr Metab Disord.

[11] Haffner D, Emma F, Eastwood DM, Duplan MB, Bacchetta J, Schnabel D (2019). Clinical practice recommendations for the diagnosis and management of X-linked hypophosphataemia. Nat Rev Nephrol.

[12] Rothenbuhler A, Fadel N, Debza Y, Bacchetta J, Diallo MT, Adamsbaum C (2019). High incidence of cranial synostosis and Chiari I malformation in children with X-linked hypophosphatemic rickets (XLHR). J Bone Miner Res.

[13] Lambert AS, Zhukouskaya V, Rothenbuhler A, Linglart A (2019). X-linked hypophosphatemia: Management and treatment prospects. Joint Bone Spine.

[14] Whyte MP, Carpenter TO, Gottesman GS, Mao M, Skrinar A, San Martin J (2019). Efficacy and safety of burosumab in children aged 1–4 years with X-linked hypophosphataemia: a multicentre, open-label, phase 2 trial. Lancet Diabetes Endocrinol.

[15] Padidela R, Whyte MP, Glorieux FH, Munns CF, Ward LM, Nilsson O (2021). Patient-reported outcomes from a randomized, active-controlled, open-label, phase 3 trial of burosumab versus conventional therapy in children with X-linked hypophosphatemia. Calcif Tissue Int.

[16] Che H, Roux C, Etcheto A, Rothenbuhler A, Kamenicky P, Linglart A (2016). Impaired quality of life in adults with X-linked hypophosphatemia and skeletal symptoms. Eur J Endocrinol.

[17] Forestier-Zhang L, Watts L, Turner A, Teare H, Kaye J, Barrett J (2016). Health-related quality of life and a cost-utility simulation of adults in the UK with osteogenesis imperfecta, X-linked hypophosphatemia and fibrous dysplasia. Orphanet J Rare Dis.

[18] Ministry of Health Consumer Affairs and Social Welfare. National Statistics Institute. Spain National Health Survey 2011–2012. Available from: https://www.sanidad.gob.es/estadEstudios/estadisticas/encuestaNacional/encuestaNac2011/PresentacionENSE2012.pdf.

[19] Skrinar A, Dvorak-Ewell M, Evins A, Macica C, Linglart A, Imel EA (2019). The lifelong impact of X-linked hypophosphatemia: results from a burden of disease survey. J Endocr Soc.

[20] Gusi N, Pérez-Sousa MA, Gozalo-Delgado M, Olivares PR (2014). Validity and reliability of the spanish EQ-5D-Y proxy version. An Pediatr (Barc).

[21] Theodore-Oklota C, Bonner N, Spencer H, Arbuckle R, Chen CY, Skrinar A (2018). Qualitative research to explore the patient experience of X-linked hypophosphatemia and evaluate the suitability of the BPI-SF and WOMAC® as clinical trial end points. Value Health.

[22] Noyes J, Edwards RT (2011). EQ-5D for the assessment of health-related quality of life and resource allocation in children: a systematic methodological review. Value Health.

[23] López-Bastida J, Peña-Longobardo LM, Aranda-Reneo I, Tizzano E, Sefton M, Oliva-Moreno J (2017). Social/economic costs and health-related quality of life in patients with spinal muscular atrophy (SMA) in Spain. Orphanet J Rare Dis.

[24] Secnik K, Matza LS, Cottrell S, Edgell E, Tilden D, Mannix S (2005). Health state utilities for childhood attention-deficit/hyperactivity disorder based on parent preferences in the United Kingdom. Med Decis Mak.

[25] Matza LS, Secnik K, Mannix S, Sallee FR (2005). Parent-proxy EQ-5D ratings of children with attention-deficit hyperactivity disorder in the US and the UK. Pharmacoeconomics.

[26] Vandewalle B, Amorim M, Ramos D, Azevedo S, Alves I, Francisco T (2021). Value-based decision-making for orphan drugs with multiple criteria decision analysis: burosumab for the treatment of X-linked hypophosphatemia. Curr Med Res Opin.

[27] Sequeira AR, Mentzakis E, Archangelidi O, Paolucci F (2021). The economic and health impact of rare diseases: a meta-analysis. Health Policy Technol.

[28] Cheung M, Rylands AJ, Williams A, Bailey K, Bubbear J (2021). Patient-reported complications, symptoms, and experiences of living with X-linked hypophosphatemia across the life-course. J Endocr Soc..

[29] Jandhyala R (2021). Neutral theory: applicability and neutrality of using generic health-related quality of life tools in diseases or conditions where specific tools are available. BMC Med Res Methodol.

[30] Jandhyala R (2022). Concordance between the schedule for the evaluation of individual quality of life-direct weighting (SEIQoL-DW) and the EuroQoL-5D (EQ-5D) measures of quality of life outcomes in adults with X-linked hypophosphatemia. Orphanet J Rare Dis.

[31] Chesher D, Oddy M, Darbar U, Sayal P, Casey A, Ryan A (2018). Outcome of adult patients with X-linked hypophosphatemia caused by *PHEX* gene mutations. J Inherit Metab Dis.

[32] Broseta JJ, López LC, Guillén E, Hernández-Jaras J (2018). Impacto en la calidad de vida y otras variables clínicas del adulto con raquitismo hipofosfatémico ligado al cromosoma X (XLH). Nefrologia.

[33] Carpenter TO, Whyte MP, Imel EA, Boot AM, Hogler W, Linglart A (2018). Burosumab therapy in children with X-linked hypophosphatemia. N Engl J Med.

[34] Imel EA, Glorieux FH, Whyte MP, Munns CF, Ward LM, Nilsson O (2019). Burosumab versus conventional therapy in children with X-linked hypophosphataemia: a randomised, active-controlled, open-label, phase 3 trial. Lancet.

[35] Insogna KL, Briot K, Imel EA, Kamenicky P, Ruppe MD, Portale AA (2018). A randomized, double-blind, placebo-controlled, phase 3 trial evaluating the efficacy of burosumab, an anti-FGF23 antibody, in adults with X-linked hypophosphatemia: week 24 primary analysis. J Bone Miner Res.

[36] Portale AA, Carpenter TO, Brandi ML, Briot K, Cheong HI, Cohen-Solal M (2019). Continued beneficial effects of burosumab in adults with X-linked hypophosphatemia: results from a 24-week treatment continuation period after a 24-week double-blind placebo-controlled period. Calcif Tissue Int.

[37] Insogna KL, Rauch F, Kamenicky P, Ito N, Kubota T, Nakamura A (2019). Burosumab improved histomorphometric measures of osteomalacia in adults with X-linked hypophosphatemia: a phase 3, single-arm, international trial. J Bone Miner Res.

[38] Briot K, Portale AA, Brandi ML, Carpenter TO, Cheong HI, Cohen-Solal M (2021). Burosumab treatment in adults with X-linked hypophosphataemia: 96-week patient-reported outcomes and ambulatory function from a randomised phase 3 trial and open-label extension. RMD Open.

[39] Skrinar A, Theodore-Oklota C, Bonner N, Arbuckle R, Williams A, Nixon A, editors. PRO152 Confirmatory psychometric validation of the Western Ontario McMaster Universities Osteoarthritis Inventory (WOMAC) in adult X-linked hypophosphatemia (XLH). ISPOR Conference; 2019 November 2–6; Copenhagen, Denmark

[40] Skrinar A, Theodore-Oklota C, Bonner N, Arbuckle R, Williams A, Nixon A, editors. PRO154 Confirmatory Psychometric Validation of the Brief Pain Inventory (BPI-SF) in adult X-linked hypophosphatemia (XLH). ISPOR Conference; 2019 November 2–6; Copenhagen, Denmark.

[41] Nixon A, Williams A, Theodore-Oklota C, editors. PRO80 Psychometric Validation of the Brief Fatigue Inventory (BFI) in adult X-linked hypophosphatemia (XLH). ISPOR Conference; 2020 May 18–20; Virtual Meeting.

[42] Badia X, Roset M, Montserrat S, Herdman M, Segura A (1999). The Spanish version of EuroQol: a description and its applications. European Quality of Life scale. Med Clin.

[43] Hernández G, Garin O, Pardo Y, Vilagut G, Pont A, Suárez M (2018). Validity of the EQ-5D-5L and reference norms for the Spanish population. Qual Life Res.

[44] EuroQol Research Foundation. EQ-5D-3L User Guide. Basic information on how to use the EQ-5D-3L instrument. Rotterdam, The Netherlands [cited 2022 June 1st]. Available from: https://euroqol.org/wp-content/uploads/2018/12/EQ-5D-3L-User-Guide_version-6.0.pdf.

