# Supplementary material for: Health-related quality of life of X-linked hypophosphatemia in Spain
Source: Orphanet J Rare Dis. 2022 Jul 29;17:298. doi: 10.1186/s13023-022-02452-0 (PMC9336088; doi:10.1186/s13023-022-02452-0)
Supplement: Supplementary file 1 — Additional file 1. Additional Tables. [file 13023_2022_2452_MOESM1_ESM.docx]

**ONLINE SUPPLEMENTARY INFORMATION**

**Table S1. Quality of life in adults with XLH and in the general Spanish population according to the 2011-2012 National Health Survey. Response percentages in the different dimensions of the EQ-5D-5L questionnaire***

| **Dimension** | **Adult patients with XLH**  **(%) (n=29)** | **General population (%) (n=20,587)** |
| --- | --- | --- |
| Mobility |  |  |
| No problems | 6.90 | 85.72 |
| Slight problems | 37.93 | 6.25 |
| Moderate problems | 31.03 | 4.76 |
| Severe problems | 20.69 | 2.45 |
| Unable to walk about | 3.45 | 0.82 |
| Self-care |  |  |
| No problems | 48.28 | 93.78 |
| Slight problems | 31.03 | 2.52 |
| Moderate problems | 17.24 | 1.77 |
| Severe problems | 3.45 | 0.93 |
| Unable to wash or dress | 0 | 1.01 |
| Usual activities |  |  |
| No problems | 20.69 | 88.86 |
| Slight problems | 34.48 | 4.87 |
| Moderate problems | 31.03 | 3.22 |
| Severe problems | 13.79 | 1.58 |
| Unable to do usual activities | 0 | 1.46 |
| Pain/discomfort |  |  |
| No pain or discomfort | 13.79 | 75 |
| Slight pain or discomfort | 10.34 | 12.51 |
| Moderate pain or discomfort | 34.48 | 8.86 |
| Severe pain or discomfort | 37.93 | 4 |
| Extreme pain or discomfort | 3.45 | 0.41 |
| Anxiety/depression |  |  |
| Not anxious or depressed | 34.48 | 84.97 |
| Slightly anxious or depressed | 31.03 | 8.6 |
| Moderately anxious or depressed | 24.14 | 4.25 |
| Severely anxious or depressed | 10.34 | 1.68 |
| Extremely anxious or depressed | 0 | 0.41 |

*Results are expressed only as percentages to simplify table layout.

**Table S2. Quality of life in adults with XLH (n= 29) and caregivers (n=21). Response percentages in the different dimensions of the EQ-5D-5L questionnaire**

| **Dimension** | **Adult patients with XLH**  **n (%)** | **Caregivers**  **n (%)** |
| --- | --- | --- |
| Mobility |  |  |
| No problems | 2 (6.90%) | 8 (38.09%) |
| Slight problems | 11 (37.93%) | 10 (47.62%) |
| Moderate problems | 9 (31.03%) | 3 (14.29%) |
| Severe problems | 6 (20.69%) | 0 (0%) |
| Unable to walk about | 1 (3.45%) | 0 (0%) |
| Self-care |  |  |
| No problems | 14 (48.28%) | 18 (85.71%) |
| Slight problems | 9 (31.03%) | 2 (9.52%) |
| Moderate problems | 5 (17.24%) | 1 (4.76%) |
| Severe problems | 1 (3.45%) | 0 (0%) |
| Unable to wash or dress | 0 (0%) | 0 (0%) |
| Usual activities |  |  |
| No problems | 6 (20.69%) | 11 (52.38%) |
| Slight problems | 10 (34.48%) | 6 (28.57%) |
| Moderate problems | 9 (31.03%) | 3 (14.29%) |
| Severe problems | 4 (13.79%) | 1 (4.76%) |
| Unable to do usual activities | 0 (0%) | 0 (0%) |
| Pain/discomfort |  |  |
| No pain or discomfort | 4 (13.79%) | 10 (52.38%) |
| Slight pain or discomfort | 3 (10.34%) | 8 (38.09%) |
| Moderate pain or discomfort | 10 (34.48%) | 2 (9.52%) |
| Severe pain or discomfort | 11 (37.93%) | 1 (4.76%) |
| Extreme pain or discomfort | 1 (3.45%) | 0 (0%) |
| Anxiety/depression |  |  |
| Not anxious or depressed | 10 (34.48%) | 14 (66.66%) |
| Slightly anxious or depressed | 9 (31.03%) | 4 (19.05%) |
| Moderately anxious or depressed | 7 (24.14%) | 2 (9.52%) |
| Severely anxious or depressed | 3 (10.34%) | 1 (4.76%) |
| Extremely anxious or depressed | 0 (0%) | 0 (0%) |
